# Whole-genome sequencing of SARS-CoV-2 isolates from symptomatic and asymptomatic individuals in Tanzania

**DOI:** 10.3389/fmed.2022.1034682

**Published:** 2023-01-04

**Authors:** Shabani Ramadhani Mziray, Marco van Zwetselaar, Charles C. Kayuki, Peter M. Mbelele, Abel N. Makubi, Alex S. Magesa, Riziki M. Kisonga, Tolbert B. Sonda, Gibson S. Kibiki, George Githinji, Scott K. Heysell, Jaffu O. Chilongola, Stellah G. Mpagama

**Affiliations:** ^1^Department of Biochemistry and Molecular Biology, Kilimanjaro Christian Medical University College, Moshi, Tanzania; ^2^Kibong’oto Infectious Diseases Hospital, Sanya Juu, Tanzania; ^3^Kilimanjaro Clinical Research Institute, Moshi, Tanzania; ^4^Oxford Nanopore Technologies, Oxford, United Kingdom; ^5^Ministry of Health, Dodoma, Tanzania; ^6^The Africa Research Excellence Fund (AREF), London, United Kingdom; ^7^KEMRI-Wellcome Trust Research Programme, Kilifi, Kenya; ^8^Department of Biochemistry and Biotechnology, Pwani University, Kilifi, Kenya; ^9^Division of Infectious Diseases and International Health, University of Virginia, Charlottesville, VA, United States

**Keywords:** COVID-19, SARS-CoV-2, whole-genome sequencing, variants of concern, variant of interest, MinION, Oxford Nanopore Technologies (ONT)

## Abstract

**Background:**

Coronavirus Disease-2019 (COVID-19), caused by Severe Acute Respiratory Syndrome Coronavirus 2 (SARS-CoV-2) accounts for considerable morbidity and mortality globally. Paucity of SARS-CoV-2 genetic data from Tanzania challenges in-country tracking of the pandemic. We sequenced SARS-CoV-2 isolated in the country to determine circulating strains, mutations and phylogenies and finally enrich international genetic databases especially with sequences from Africa.

**Methods:**

This cross-sectional study utilized nasopharyngeal swabs of symptomatic and asymptomatic adults with positive polymerase chain reaction tests for COVID-19 from January to May 2021. Viral genomic libraries were prepared using ARTIC nCoV-2019 sequencing protocol version three. Whole-genome sequencing (WGS) was performed using Oxford Nanopore Technologies MinION device. *In silico* genomic data analysis was done on ARTIC pipeline version 1.2.1 using ARTIC nCoV-2019 bioinformatics protocol version 1.1.0.

**Results:**

Twenty-nine (42%) out of 69 samples qualified for sequencing based on gel electrophoretic band intensity of multiplex PCR amplicons. Out of 29 isolates, 26 were variants of concern [Beta (*n* = 22); and Delta (*n* = 4)]. Other variants included Eta (*n* = 2) and B.1.530 (*n* = 1). We found combination of mutations (S: D80A, S: D215G, S: K417N, ORF3a: Q57H, E: P71L) in all Beta variants and absent in other lineages. The B.1.530 lineage carried mutations with very low cumulative global prevalence, these were nsp13:M233I, nsp14:S434G, ORF3a:A99S, S: T22I and S: N164H. The B.1.530 lineage clustered phylogenetically with isolates first reported in south-east Kenya, suggesting regional evolution of SARS-CoV-2.

**Conclusion:**

We provide evidence of existence of Beta, Delta, Eta variants and a locally evolving lineage (B.1.530) from samples collected in early 2021 in Tanzania. This work provides a model for ongoing WGS surveillance that will be required to inform on emerging and circulating SARS-CoV-2 diversity in Tanzania and East Africa.

## 1. Introduction

Coronavirus disease 2019 (COVID-19) is an emerging viral disease caused by severe acute respiratory syndrome coronavirus 2 (SARS-CoV-2), which belongs to *Coronaviridae* family (1). An outbreak of the disease was reported for the first time in December 2019 in Wuhan city, Hubei province, China. The disease rapidly spread to other regions with sky-rocketing numbers of new infections. On 11 March 2020, the World Health Organization (WHO) declared COVID-19 a pandemic (2). By 25 November 2022, a total of 636,440,663 confirmed cases of COVID-19 were reported globally to the WHO and 6,606,624 (∼1%) deaths cumulatively whereas Africa contributed 9, 390,554 cases and 174,993 (∼2%) cumulative deaths (3). Tanzania described the existence of the first case of COVID-19 on 16 March 2020 (4). The country encountered several waves of COVID-19 pandemic and as of 25 November 2022, confirmed cases of COVID-19 were 40,471 with 845 (∼2%) cumulative deaths (3 ). The country joined the COVID-19 Vaccine Global Access (COVAX) initiative program in the mid of 2021 with limited knowledge of the molecular epidemiology of COVID-19 (5). COVID-19 vaccine is an effective means of containing the COVID-19 pandemic, however, antigenic drift particularly at the antigenic epitope and receptor binding sites may interfere with the compatibility of vaccine and field strains (6).

SARS-CoV-2 is an enveloped positive sense, single-stranded RNA virus with a genomic RNA size of approximately 30 Kb (7). The viral genome has two major parts: the open reading frame 1a and b (ORF1a and ORF1b) toward the 5′ end of the genome, and the structural protein encoding region toward the 3′ end. ORF1 encodes 16 non-structural proteins that are responsible for the formation of the replication-transcription complex including an RNA dependent RNA polymerase (RdRp). The structural protein encoding region translates into the Spike (S), Envelope (E), Membrane (M) and Nucleocapsid (N) proteins of the virion (7). The S protein allows viral entry into the host cell by binding to the angiotensin-converting enzyme 2 receptors and is a common vaccine target (8). The genome of SARS-CoV-2 is prone to mutations that allow emergence of different variants including the Variants of Concern (VOC). The strains assigned as VOC have high transmissibility and/or pathogenicity. Importantly, VOC can be associated with reduced vaccine and therapeutic effectiveness, as well as impaired detection by the currently approved diagnostics (9).

Whole-genome sequencing (WGS) of SARS-CoV-2 can address certain polymerase chain reaction (PCR) based diagnostic limitations, and additionally describe the specific SARS-CoV-2 variant, or when applied across larger populations of isolates, also inform transmission or evolutionary dynamics. Capacity for WGS and complementary bioinformatics is not widely available in Tanzania, a problem shared by some other countries similarly burdened by the COVID-19 pandemic and a lack of early coordinated scientific response. For example, since Tanzania announced the first case of COVID-19 (4), and while routine testing of asymptomatic travelers was eventually administered, routine availability of testing symptomatic individuals was never fully scaled to meet the population needs (10). Apart from 44 travel cases and 9 apparent travel cases, no SARS-COV-2 sequences from Tanzania had been deposited in public repositories prior to this publication. Consequently, this study aimed to perform WGS of SARS-CoV-2 from a convenient but representative sample set in Tanzania to describe circulating variants, mutations and phylogenies, while developing a model for future response.

## 2. Materials and methods

### 2.1. Study design and population

This retrospective cross-sectional study was done from January to May 2021 to characterize the whole-genomes of SARS-CoV-2 isolates from archived nasopharyngeal swabs. The samples were collected from symptomatic and asymptomatic individuals with PCR positive test for COVID-19 from three diverse regions in Tanzania. Symptomatic case was regarded as an individual who had tested positive for SARS-CoV-2 using nucleic acid amplification test with at least one of the following signs and/or symptoms; -fever, cough, tiredness, loss of taste or smell, sore throat, headache, aches and pains, diarrhea, a rash on skin, or discoloration of fingers or toes, red or irritated eyes, difficulty breathing or shortness of breath, loss of speech or mobility or confusion, chest pain. Whereas, asymptomatic cases were individuals who tested positive for SARS-CoV-2 using a nucleic acid amplification test but had no symptoms that were consistent with COVID-19 and were largely from travelers (11).

### 2.2. Settings, sample collection, and management

**FIGURE 1:**
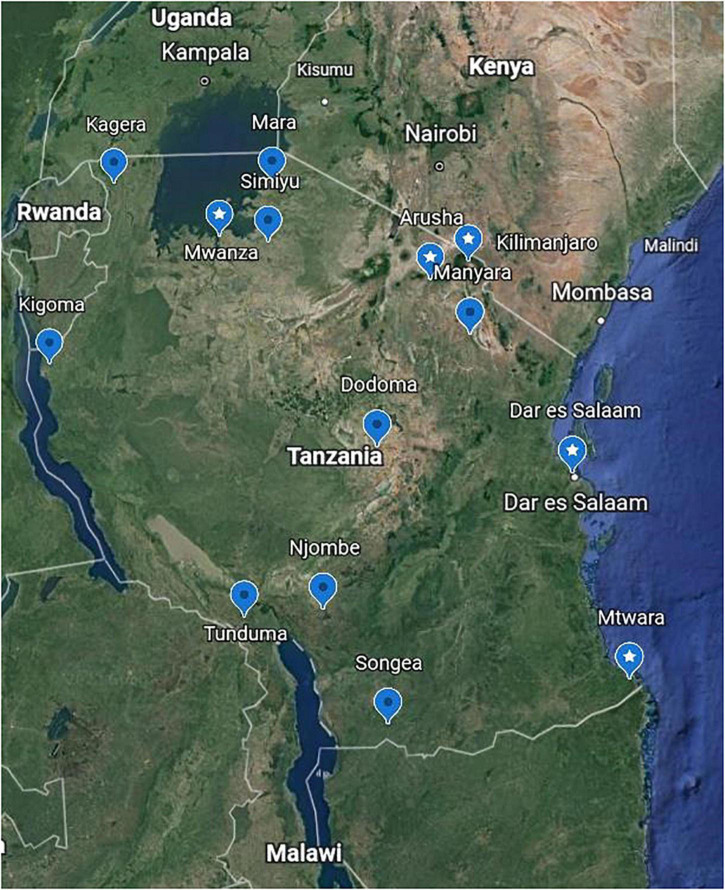
Sample collection sites. A Google Earth map showing the locations in Tanzania where 69 nasopharyngeal swabs were collected from individuals confirmed with coronavirus disease-2019 (COVID-19) for this study. Labels with star show the locations where the 29 samples that were sequenced originated; these match the place names in Table 2.

Nasopharyngeal swabs were obtained from the bio-repository of the National Public Health Laboratory, Dar es Salaam, Tanzania and shipped to Kibong’oto Infectious Diseases Hospital (KIDH) Laboratory, northern Tanzania, for SARS-CoV-2 sequencing. Briefly, within the biorepository, we sorted all the archived nasopharyngeal swabs collected from regions near neighboring countries as well as administrative regions with international airports (Mwanza, Dodoma, Kilimanjaro and Dar es Salaam) (Figure 1). Thereafter, we randomly selected 69 COVID-19 PCR positive (<30 cycle threshold values) nasopharyngeal swabs. These specimens were previously collected from symptomatic and asymptomatic travelers and from patients seeking medical care in health facilities in Tanzania. The nasopharyngeal swabs were kept in virus transport medium, and were shipped at 2–8°C to KIDH laboratory. Upon arrival at KIDH laboratory, the samples were immediately stored at −80°C until viral RNA extraction, genomic library preparation and sequencing. SARS-CoV-2 PCR was not repeated at KIDH laboratory.

### 2.3. Laboratory procedures

#### 2.3.1. SARS-CoV-2 RNA extraction

Severe acute respiratory syndrome coronavirus-2 RNA was extracted from 100 μL of nasopharyngeal swabs using *Quick*-RNA Viral kit (Zymo Research Corp., CA, United States) as per manufacturer’s protocol. Briefly, the viruses in the swabs were inactivated by mixing equal volumes of specimen and that of DNA/RNA shield (2X concentrate). Viral RNA purification was conducted using Zymo-Spin™ IC columns, whereby the RNA was allowed to bind into the columns and washed by viral wash buffer and absolute ethanol (99.9%). The final eluted volume of viral RNA from each sample was 15 μL.

#### 2.3.2. Genomic library preparation

Genomic library preparation was carried out using the ARTIC nCoV-2019 sequencing protocol v3 (LoCost) (12). Briefly, 2 μL of LunaScript RT SuperMix (5X) (New England BioLabs, MA, United States) was used to generate complementary DNA (cDNA) from 8 μL of the extracted viral RNA. No-RT control mix (5X) and a negative control (nuclease-free water) were included in the cDNA synthesis step to monitor cross-contamination between samples in the downstream PCR amplifications. Using Q5 Hot Start High-Fidelity 2X Master Mix (New England BioLabs, MA, United States), reaction 1 and 2 of multiplex PCR was set to amplify 2.5 μL of cDNA in combination with 10 μM ARTIC nCoV-2019 V3 primer pool 1 and 2, respectively. Primer pool V3 with added primer pair 74_alt0 (Inqaba Biotec, Pretoria, South Africa) was later applied to reduce primer dropout (failure of the ARTIC v3 primers to hybridize the targeted SARS-CoV-2 genome site resulting into incomplete genome sequence coverage) in multiplex PCR. The programs for multiplex PCR were: Heat activation at 98°C, 30 s; Denaturation at 95°C, 15 s, 35 cycles; Annealing/Extension at 63°C, 5 min, 35 cycles; and Hold at 4°C. Gel (1.5%) electrophoresis was done at 100V for 20 min to assess the quality of amplicons from each of the two multiplex PCR reactions. Equal volumes of the amplified cDNA from the two reactions were combined together followed by 1:10 dilution using nuclease-free water. From this, 3.3 μL of the diluted PCR amplicons was end-prepared using NEBNext Ultra II End Repair/dA-tailing module (New England BioLabs, MA, United States) by incubation at 20°C for 15 min and 65°C for 15 min. From the end-preparation step, 0.75 uL of the end-prepared reaction mixture was barcoded using Native Barcoding Expansion Kit 1–12 and 13–24 (ONT, Oxford, United Kingdom) at 20°C for 20 min and 65°C for 10 min incubation. The barcoded cDNA were pooled together into 1.5 mL eppendorf tube. Size selection of the pooled-barcoded cDNA with 400 bp was done using 0.4× Ampure XP beads (Beckman Coulter, IN, United States). Resuspension of the beads was done twice using 250 μL of short fragment buffer (ONT, Oxford, United Kingdom) followed by a single bathing of the pellets using 70% ethanol. Elution of the pooled-barcoded cDNA was done using 30 μL of nuclease-free water. Adapter Mix II (ONT, Oxford, United Kingdom) were ligated on 30 μL of pooled-barcoded cDNA by Quick T4 DNA ligase (New England BioLabs, MA, United States). The ligated libraries were washed twice using 250 μL of short fragment buffer (ONT, Oxford, United Kingdom) to remove excess adapters. Finally, 15 μL of the library was eluted from the beads by elution buffer (ONT, Oxford, United Kingdom). Quantification of the libraries was done using Qubit Fluorometer v2 (Invitrogen, Thermo Fisher Scientific, MA, United States).

#### 2.3.3. Sequencing

The eluted genomic libraries were loaded into R9.4.1 flow cell (ONT, Oxford, United Kingdom) and sequenced using MinION Mk1B sequencing device (ONT, Oxford, United Kingdom). The MinKNOW software (ONT, Oxford, United Kingdom) was used to start and monitor the progress of the sequencing run. Mapped reads of SARS-CoV-2 were visualized using Read Assignment, Mapping, and Phylogenetic Analysis in Real Time (RAMPART) v1.2.0 (ARTIC).

#### 2.3.4. SARS-CoV-2 sequences quality and genome assembly

The ARTIC nCoV-2019 bioinformatics protocol v1.1.0 (13) was used with ARTIC pipeline v1.2.1 to carry out *in silico* analysis of SARS-CoV-2 sequences. Briefly, basecalling was done with Guppy 5.0.7 using the super high accuracy (sup) model. Demultiplexing was done with Guppy barcoder 5.0.7. Basecalling and demultiplexing were later repeated with Guppy 6.0.1, with no changes in output. Read filtering was performed with guppyplex, selecting for lengths 300–700 bp. Reads were assembled and polished, and consensus sequences produced by the ARTIC MinION 1.2.0 pipeline using Medaka (model r941_min_high_g360, strict, no normalization). Multiple alignments of the consensus sequences were performed with MAFFT v7.475 (14) (globalpair, 5000 iterations). The reference genome was hCoV-19/Wuhan/WIV04/2019 (WIV04, GenBank accession *MN908947.3*). In-house tools were used to obtain read and base counts, Q7 percentage, and genome coverage. NextClade Web 1.10.0 (15) with default settings was used to assess sequence quality and amplicon drop-outs.

#### 2.3.5. SARS-CoV-2 lineage and clade assignment

The consensus sequences were assigned PANGO lineages (16) and WHO designations using Pangolin version 3.0.3 with Pango LEARN version 2021-11-25 and designation version 1.2.101 (17). NextClade Web 1.10.0 (15) was used to assign clades and WHO designations. Single nucleotide polymorphisms (SNPs) were obtained from the ARTIC pipeline “pass” variant call format output, translated to genomic loci using sc2calc (18), and validated using CoV-GLUE 0.1.18 (19). CoVsurver enabled by GISAID (20) and mutation tracker in outbreak.info (21) were used to obtain prevalence and etiology information for mutations. NextClade Web 1.10.0 (15) output was used to visualize the phylogenetic placement of the isolates in NextStrain Auspice 2.32.0 (22). Assignment to a tree node was based on the genetic distance of an isolate to other isolates, where distance was measured in terms of the number of mutations that separate pairs of isolates. Tree branches with fewer nodes were sorted toward the top of the tree using default settings of Interactive Tree Of Life (iTOL) v5 (23). Also, iTOL v5 was used to make tree branch labels, alignment, leaf node symbols and other manipulations. To facilitate interaction with big phylogenetic trees, the files were kept in json files for visualization with auspice web-based tool available at *https://auspice.us/?d=tree&p=full.*

### 2.4. Ethical considerations

The study protocol was reviewed and approved by Kilimanjaro Christian Medical University College research ethics review committee. Permission to conduct the study was granted by authorities of the KIDH and National Public Health Laboratory. Individual consent from participants was deemed not necessary for use of the archived and previously PCR-tested samples. The results of SARS-CoV-2 sequencing were shared with the Tanzanian Ministry of Health to inform policy actions.

## 3. Results

### 3.1. Quality of PCR amplicons and sequence reads

Out of 69 individuals’ nasopharyngeal swabs, 40 (58%) showed faint or no bands in 1.5% agarose gel electrophoresis of the amplified SARS-CoV-2 cDNA, suggesting that RNA was degraded and were therefore removed from downstream library preparations. No bands were observed in No-RT control and negative control after 1.5% agarose gel electrophoresis, indicating absence of cross-contamination between samples during conduction of the wet laboratory procedures. The remaining 29 (42%) samples showed strong bands in the gel electrophoresis and were sequenced. Percentage of bases called with Q-score ≥7 (80% accuracy) ranged from 89.2 to 92.7%, while percentage GC contents ranged from 38.6 to 40.8%. The median read-depth was 2317×, IQR [1332–3241] (Table 1). Genome coverage, however, was mediocre (90.0–95.2%) for 15 samples, to poor (78.3–89.9%) for 14 samples, due to amplicon drop-out (Supplementary File 1).

**TABLE 1:** Quality of sequence reads and metrics of assembled genomes.

Sample ID	Filtered sequence quality				Assembled genome metrics				
	N_read	N_bases	% Q7	% gc	Av.depth	Bases	Acgt	Ns	%_coverage
Tanzania/KIDH-01B01/2021	166095	82564938	89.2	38.6	2761.09	29885	28117	1768	94
Tanzania/KIDH-01B03/2021	161991	80777384	89.4	39.7	2701.31	29885	28313	1572	94.7
Tanzania/KIDH-01B04/2021	180774	89911765	89.3	38.7	3006.78	29885	28040	1845	93.8
Tanzania/KIDH-01B06/2021	74606	36924711	89.2	39.6	1234.82	29903	27507	2396	91.9
Tanzania/KIDH-01B10/2021	195836	97310663	89.4	39.1	3254.21	29885	28117	1768	94
Tanzania/KIDH-02B01/2021	135793	69293557	92.5	39.2	2317.28	29890	26180	3710	87.5
Tanzania/KIDH-02B02/2021	202942	103953409	92.4	39.2	3476.35	29885	28427	1458	95.1
Tanzania/KIDH-02B04/2021	210798	107844465	92.7	38.7	3606.48	29885	28437	1448	95.1
Tanzania/KIDH-02B05/2021	188853	96525895	92.6	39.2	3227.97	29885	27858	2027	93.2
Tanzania/KIDH-02B06/2021	257911	132139681	92.6	39	4418.94	29885	27877	2008	93.2
Tanzania/KIDH-02B07/2021	133997	68354715	92.8	39.3	2285.88	29894	26755	3139	89.4
Tanzania/KIDH-02B08/2021	41196	21041845	92.3	39.8	703.67	29894	23564	6330	78.8
Tanzania/KIDH-02B09/2021	205184	104823135	92.6	39.7	3505.44	29894	27050	2844	90.4
Tanzania/KIDH-02B10/2021	169106	86409963	92.7	39.1	2889.68	29885	28084	1801	93.9
Tanzania/KIDH-02B11/2021	53943	27476577	92.4	39.7	918.857	29887	23403	6484	78.3
Tanzania/KIDH-02B12/2021	178378	91090774	92.4	39	3046.21	29894	27272	2622	91.2
Tanzania/KIDH-02B14/2021	215061	109945776	92.5	39.8	3676.75	29890	28118	1772	94
Tanzania/KIDH-02B15/2021	127156	64963955	92.5	40.2	2172.49	29890	26475	3415	88.5
Tanzania/KIDH-03B01/2021	109621	55334816	91.2	39.6	1850.48	29894	25686	4208	85.9
Tanzania/KIDH-03B03/2021	164903	83821018	91.4	40	2803.1	29894	27327	2567	91.4
Tanzania/KIDH-03B04/2021	64903	32939529	91.3	39.3	1101.55	29894	25384	4510	84.9
Tanzania/KIDH-03B05/2021	80425	40830649	91.4	39.9	1365.44	29896	25326	4570	84.7
Tanzania/KIDH-03B07/2021	108916	55223677	91.6	39.7	1846.76	29894	26204	3690	87.6
Tanzania/KIDH-03B10/2021	62441	31639518	91.3	40	1058.07	29887	25027	4860	83.7
Tanzania/KIDH-03B11/2021	36176	18369335	91	39.9	614.297	29894	24621	5273	82.3
Tanzania/KIDH-03B12/2021	133061	67459583	91	39.6	2255.95	29885	26811	3074	89.7
Tanzania/KIDH-03B13/2021	372353	188965831	91.3	39.3	6319.29	29885	28437	1448	95.1
Tanzania/KIDH-03B14/2021	134099	68069456	91.1	40	2276.34	29894	26768	3126	89.5
Tanzania/KIDH-03B18/2021	45350	22894416	91.1	40.8	765.623	29894	24577	5317	82.2

Samples with ID containing KIDH-01 used ARTIC primer pool V3; samples with ID containing KIDH-02 or KIDH-03 used ARTIC primer pool V3 with added primer pair 74_alt0.

### 3.2. Characteristics of sequenced samples

Demographic and clinical characteristics of the 29 sequenced samples are presented in Table 2. Most samples (*n* = 17) were collected in April 2021, when the presumed “Beta wave” of the pandemic had subsided. Seventeen samples were collected in Dar es Salaam, the most populous city in Tanzania. Samples were collected from symptomatic individuals suspected to have COVID-19 (*n* = 17) and from asymptomatic travelers (*n* = 12). Out of 17 symptomatic individuals, 5 were hospitalized in January 2021 at tertiary hospitals in Dar es Salaam.

**TABLE 2:** Characteristics of sequenced samples (*n* = 29).

Sample ID	Age (years)	Sex	Date of sample collection	Place of sample collection	Recent travel history	Category	ϮClinical status	Assigned PANGO lineage	WHO label
Tanzania/KIDH-01B01/2021	Unknown	Female	23-January-21	Dar es Salaam	Unknown	*Suspect	Symptomatic	B.1.351	Beta
Tanzania/KIDH-01B03/2021	Unknown	Female	23-January-21	Dar es Salaam	Unknown	*Suspect	Symptomatic	B.1.351	Beta
Tanzania/KIDH-01B04/2021	Unknown	Male	23-January-21	Dar es Salaam	Unknown	*Suspect	Symptomatic	B.1.351	Beta
Tanzania/KIDH-01B06/2021	Unknown	Male	23-January-21	Dar es Salaam	Unknown	*Suspect	Symptomatic	B.1.530	–
Tanzania/KIDH-01B10/2021	Unknown	Male	23-January-21	Dar es Salaam	Unknown	*Suspect	Symptomatic	B.1.351	Beta
Tanzania/KIDH-02B01/2021	Unknown	Unknown	25-April-21	Mwanza	Unknown	Suspect	Symptomatic	B.1.617.2	Delta
Tanzania/KIDH-02B02/2021	Unknown	Unknown	26-April-21	Dar es Salaam	Unknown	Traveler	Asymptomatic	B.1.351	Beta
Tanzania/KIDH-02B04/2021	Unknown	Unknown	26-April-21	Dar es Salaam	Unknown	Suspect	Asymptomatic	B.1.351	Beta
Tanzania/KIDH-02B05/2021	Unknown	Unknown	22-April-21	Dar es Salaam	Kenya	Traveler	Asymptomatic	B.1.351	Beta
Tanzania/KIDH-02B06/2021	Unknown	Unknown	30-March-21	Mwanza	Philippines	Traveler	Asymptomatic	B.1.351	Beta
Tanzania/KIDH-02B07/2021	Unknown	Unknown	5-April-21	Dar es Salaam	Unknown	Traveler	Asymptomatic	B.1.351	Beta
Tanzania/KIDH-02B08/2021	Unknown	Unknown	12-April-21	Dar es Salaam	Zambia	Traveler	Asymptomatic	B.1.351	Beta
Tanzania/KIDH-02B09/2021	85	Female	10-April-21	Kilimanjaro	Unknown	Suspect	Symptomatic	B.1.351	Beta
Tanzania/KIDH-02B10/2021	45	Male	10-April-21	Mwanza	India	Suspect	Symptomatic	B.1.351	Beta
Tanzania/KIDH-02B11/2021	55	Male	10-April-21	Mwanza	Unknown	Suspect	Symptomatic	B.1.525	Eta
Tanzania/KIDH-02B12/2021	80	Female	11-April-21	Kilimanjaro	Unknown	Suspect	Symptomatic	B.1.351	Beta
Tanzania/KIDH-02B14/2021	Unknown	Unknown	3-May-21	Dar es Salaam	India	#Traveler	Asymptomatic	B.1.617.2	Delta
Tanzania/KIDH-02B15/2021	Unknown	Unknown	3-May-21	Dar es Salaam	India	#Traveler	Asymptomatic	B.1.617.2	Delta
Tanzania/KIDH-03B01/2021	39	Male	25-April-21	Dar es Salaam	Unknown	Suspect	Symptomatic	B.1.351	Beta
Tanzania/KIDH-03B03/2021	Unknown	Unknown	26-April-21	Dar es Salaam	Unknown	Traveler	Asymptomatic	B.1.351	Beta
Tanzania/KIDH-03B04/2021	Unknown	Unknown	26-April-21	Dar es Salaam	Unknown	Traveler	Asymptomatic	B.1.351	Beta
Tanzania/KIDH-03B05/2021	27	Female	22-April-21	Mwanza	Unknown	Suspect	Symptomatic	B.1.617.2	Delta
Tanzania/KIDH-03B07/2021	66	Female	23-April-21	Mwanza	Unknown	Suspect	Symptomatic	B.1.351	Beta
Tanzania/KIDH-03B10/2021	52	Male	30-March-21	Arusha	Unknown	Suspect	Symptomatic	B.1.525	Eta
Tanzania/KIDH-03B11/2021	46	Female	30-March-21	Mtwara	Unknown	Suspect	Symptomatic	B.1.351	Beta
Tanzania/KIDH-03B12/2021	60	Male	30-March-21	Mwanza	Unknown	Suspect	Symptomatic	B.1.351	Beta
Tanzania/KIDH-03B13/2021	58	Male	30-March-21	Dar es Salaam	Unknown	Suspect	Symptomatic	B.1.351	Beta
Tanzania/KIDH-03B14/2021	Unknown	Unknown	5-April-21	Dar es Salaam	Unknown	Traveler	Asymptomatic	B.1.351	Beta
Tanzania/KIDH-03B18/2021	Unknown	Unknown	11-April-21	Mwanza	Unknown	Traveler	Asymptomatic	B.1.351	Beta

*The individuals were hospitalized in Dar es Salaam at different unknown dates. In the category column, all the travelers were outgoing except the two arriving from India indicated by #. ϮSymptomatic case was regarded as an individual who had tested positive for severe acute respiratory syndrome coronavirus-2 (SARS-CoV-2) using nucleic acid amplification test with at least one of the following signs and/or symptoms; -fever, cough, tiredness, loss of taste or smell, sore throat, headache, aches and pains, diarrhea, a rash on skin, or discoloration of fingers or toes, red or irritated eyes, difficulty breathing or shortness of breath, loss of speech or mobility or confusion, chest pain. Whereas, asymptomatic cases were individuals who tested positive for SARS-CoV-2 using a nucleic acid amplification test but had no symptoms that were consistent with COVID-19 and were largely from travelers.

### 3.3. SARS-CoV-2 variants and diversity

**FIGURE 2:**
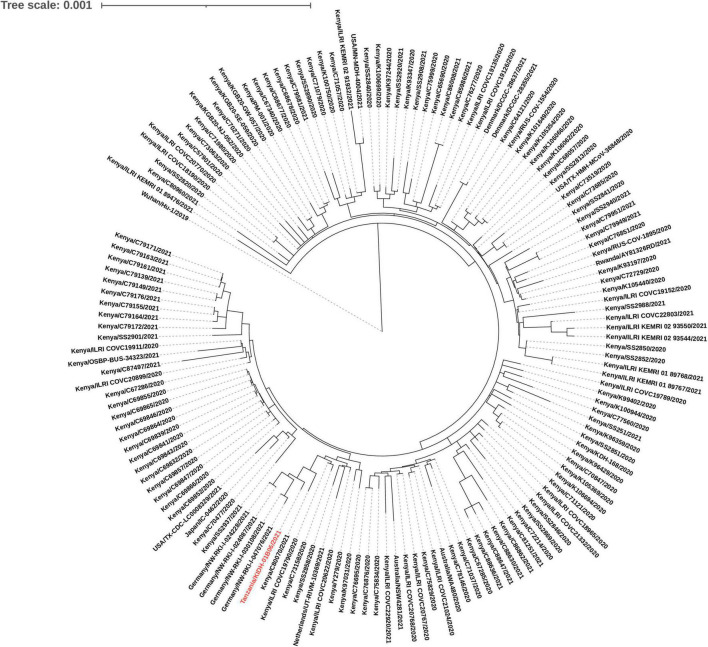
A maximum likelihood phylogenetic tree of B.1.530 lineage placed against 150 global genomes belonging to B.1.530 lineage. Isolate Tanzania/KIDH-01B06/2021 is colored red.

Out of 29 isolates sequenced, 26 were variants of concern [Beta (*n* = 22); and Delta (*n* = 4)]. Other variants included Eta (*n* = 2) and B.1.530 (*n* = 1). Two of the four Delta isolates (Tanzania/KIDH-02B14/2021 and Tanzania/KIDH-02B15/2021) were from asymptomatic travelers arriving from India of whom samples were collected on 3 May 2021 (Table 2), and were placed in NextStrain clades 21A and 21J, respectively. The other two Delta isolates (Tanzania/KIDH-03B05/2021 and Tanzania/KIDH-02B01/2021) were from local symptomatic suspects, collected in Mwanza on 22 April 2021 and 25 April 2021, respectively, and both placed in NextStrain clade 21I. Strains from all three clades of Delta variant had been detected in Africa as early as January 2021, with notably 21I prevalent in neighboring Rwanda. Isolate Tanzania/KIDH-03B05/2021 and Tanzania/KIDH-02B01/2021 of NextStrain clade 21I clustered closely with isolates from neighboring Rwanda, Uganda, Kenya, and Burundi, suggesting regional circulation (Supplementary File 2). The common ancestors of the Beta variant seem to have come from southern Africa to the rest of the world (Supplementary File 2). Based on the genomic proximity to neighboring sequences in the phylogenetic tree, most Beta isolates from Tanzania appear to descend from strains originating from bordering countries in the south and south-west Africa. Figure 2 describes genetic relatedness between Tanzania/KIDH-01B06/2021 isolate (identified by red color) with 150 global genomes belonging to lineage B.1.530 (24). The maximum likelihood phylogenetic tree shows that this isolate clustered with similar isolates identified earlier in southern Kenya in 2020. The sample date for this isolate was 23 January 2021. However, four isolates of B.1.530 identified in Germany seemed to be closely related to Tanzania/KIDH-01B06/2021 but with more diversity. Approximately 50% of B.1.530 sequence divergence was explained by date of sample collection (*r*^2^ = 0.496). Eta variant may have descent from ancestors in western Africa, where Eta was first detected in December 2020 (Supplementary File 2). Eta was a variant under investigation that is no longer circulating at a pace of global public health significance.

### 3.4. Profiles of SARS-CoV-2 mutations

All 29 (100%) SARS-CoV-2 isolates had the non-synonymous spike mutation D614G. Twenty-seven (93%) of the isolates contained mutation C14408T (ORF1ab:P4715L, nsp12:P323L) that was not identified in the two isolates belonging to the B.1.525 lineage. Twenty-four (83%) of the isolates, and a majority assigned to B.1.351 lineage, had nucleocapsid mutation T205I (C28887T) that was not identified in the four isolates assigned to B.1.617.2 lineage (Supplementary File 1).

The combination of mutations (S: D80A, S: D215G, S: K417N, ORF3a: Q57H, E: P71L) characteristic of the B.1.351 lineage was observed in all 22 B.1.351 isolates and absent in the other three lineages. The deletion of 9nt at position 22281, a common marker for B.1.351 lineage, was found only in the 22 isolates assigned B.1.351 lineage. The 9nt deletion at 11288 is common to all VOC, but was only found in 10 isolates assigned to B.1.351 lineage. Twenty-one B.1.351 isolates contained spike mutation A701V that was not observed in the other three lineages. ORF3a:S171L was seen only in 20 out of 22 B.1.351 lineages. The spike mutations L18F (*n* = 18), N501Y (*n* = 16), and nucleocapsid mutation P13S (*n* = 14) were only found in B.1.351 lineage isolates. The S: E484K mutation was shared by B.1.351 (*n* = 14) and B.1.525 (*n* = 2) isolates. Apart from B.1.351, B.1.525 lineage was the only other lineage containing the spike mutation E484K (*n* = 2) (Supplementary File 1).

The four isolates assigned to Delta variant shared ten defining non-synonymous mutations for the Delta variant (S: T19R, S: L452R, S: T478K, S: P681R, S: D950N, ORF3a:S26L, N: D63G, N: R203M, N: D377Y), and a 6nt deletion at 28248. The other signature deletion 22029:6 was found in three out of four isolates assigned to Delta variant. The signature matrix mutation M: I82T for Delta was shared with the two Eta variants. The other two defining mutations for Delta, ORF7a: V82A and ORF7a: T120I were only found in Delta isolates Tanzania/KIDH-02B14/2021 and Tanzania/KIDH-02B15/2021, collected from two arrivals from India. Other mutations that characterized the four Delta isolates were G15451A (nsp12:G671S) and C16466T (nsp13:P77L) (Supplementary File 1).

Isolate Tanzania/KIDH-01B06/2021 assigned to the B.1.530 lineage contained the fewest non-synonymous mutations (*n* = 10) overall. The highest number of mutations was 28 in Delta isolate Tanzania/KIDH-02B14/2021 (Supplementary File 1). This isolate was characterized by a unique pattern of mutations that were not found in the other 28 isolates. These include A3291G (nsp3:Q191R), G16935T (nsp13:M233I), A19339G (nsp14:S434G), G25687T (ORF3a:A99S), and spike mutations T22I (C21627T) and N164H (A22052C). All except the A3291G mutation had low cumulative global prevalence of <0.5%.

### 3.5. Nucleotide substitutions in PCR primer binding sites

All 29 genomes had at least one to a maximum of three nucleotide substitution in the primer binding site for RT-PCR test (Supplementary File 1). A total of nine nucleotide substitutions in RT-PCR targeted genomic regions were identified, whereby C28887T in N gene was commonly observed (*n* = 20). This position is in the genomic site targeted by the China CDC RT-PCR test (Table 3).

**TABLE 3:** Coronavirus disease-2019 (COVID-19) polymerase chain reaction (PCR) test kits affected by primer nucleotide substitutions.

COVID-19 PCR test kit	PCR primer target in SARS-CoV-2 genome	PCR primer type	PCR primer nucleotide substitution	Number of genomes with PCR primer nucleotide substitution
China CDC	*N*	Forward	C28887T	20
China CDC	*N*	Forward	G28881T	5
China CDC	*N*	Forward	G28882T	1
US CDC	*N1*	P	C28310T	12
US CDC	*N3*	Forward	A28699G	2
Charite	*RdRp*	Forward	G15451A	4
Charite	*E*	Forward	A26271G	3
Charite	*N*	P	C28775T	1
Pasteur	*RdRp*	Reverse	C14184T	1

N, nucleocapsid, RdRp, RNA dependent RNA polymerase.

## 4. Discussion

This study reports Beta and Delta variants of concern (VOC), as well as the Eta variant of interest isolated by whole-genome sequencing (WGS) from selected samples collected at different time points from January through May 2021 in Tanzania. Most isolates were from symptomatic individuals indicating that SARS-CoV-2 infections were in active circulation at the time of sample collection. The variants had substantial number of mutations including mutations in the primer binding sites for commonly used COVID-19 RT-PCR test kits, suggesting the potential of the SARS-CoV-2 in evading detection by RT-PCR tests. We further report lineage B.1.530 in a sample collected in January 2021 with mutations of very low global prevalence that clustered phylogenetically with isolates first identified in Kenya, suggesting that it was a locally or regionally evolving lineage.

Not only do the processes provide a model for describing SARS-CoV-2 evolution in Tanzania in the future, for the first time in the country; we report existence of Delta and Beta VOC from both symptomatic and asymptomatic individuals in the second wave of the pandemic. The VOC have increased transmissibility compared to other variants of SARS-CoV-2, and increased pathogenic potential toward severe disease (9). The increased transmissibility of VOC is conferred by the presence of the highly prevalent mutations in the spike such as D614G and N501Y that orchestrate increased expression of spike protein density that increase infectivity and enhanced angiotensin converting enzyme-2 (ACE-2) binding, respectively (25–27). Spike mutation L452R, and T478K were also implicated in causing increased transmissibility, infectivity and pathogenicity of SARS-CoV-2 by immune evasion and strong affinity of the viruses to cells through ACE-2 (28, 29). In addition, spike mutation P681R was associated with increased viral replication and transmissibility of Delta variant (30). The potential of the VOC, particularly Delta, to spread from asymptomatic individuals was documented previously and this warrants additional measures such as contact tracing in order to curb the spread of the virus (31). Apart from that, the VOC can evade neutralizing antibodies after vaccination, as well as detection by the currently approved diagnostics. These attributes of the VOC are driven partly by the presence of mutations such as E484K in the spike and other targets of the vaccines (32). Likewise, spike K417N is associated with evasion from neutralizing antibody binding although not to a large extent compared to spike E484K mutation (33).

Our finding of a regionally evolving lineage, B.1.530, that was first identified in Kenya in October 2020, is of further importance (34). The lineage showed unique profiles of mutations such as S: N164H, S: T22I, nsp14:S323G, and nsp13:M233I with low cumulative global prevalence and undocumented phenotypes (35). The shared mutation nsp14:S323G with isolates of the same lineage in southern Kenya suggested that the lineage was evolving in the region. It also had the nsp13:M233I substitution that was previously reported in 5 countries, with <0.5% cumulative global prevalence (36). Likewise, we highlight mutation nsp14:S434G that had also very low global prevalence, with 43 reports from southern Kenya: Taita Taveta (*n* = 28), Kilifi (*n* = 7), and Mombasa (*n* = 8) as of October 2020 (34). Although the prevalence of the lineage appears to decline in the wake of the Omicron variant, further research to translate these mutations with low global prevalence to phenotypic characteristics may be particularly important in Tanzania where there may be some ongoing regional circulation.

Unexpectedly, we found that all the 29 SARS-CoV-2 genomes had at least one nucleotide substitution in primer binding sites for RT-PCR. Presence of such substitutions increases the potential of variants to evade detection by RT-PCR diagnostic tests, as documented earlier in the pandemic (37). Mutations in the RT-PCR primer binding sites contributed to false negative RT-PCR tests (38), a driver for underestimation of the true burden of COVID-19 infections as well as reduced prompt clinical and epidemiological interventions (39). Periodic evaluation of the performance characteristics of COVID-19 RT-PCR test kits by sequencing the primer/probe binding sites is warranted. The choice of RT PCR diagnostic test kits with multiple gene targets for SARS-CoV-2 may also increase recovery rates of the virus in the given specimens from Tanzania or other regions where this finding is common (40). Alternatively, two different diagnostic kits may be used in the laboratories to minimize false negative results.

## 5. Conclusion

Use of WGS provided evidence of circulation of Beta, Delta, and Eta variants and several regionally evolving variants with signature mutations circulating in Tanzania in early 2021. The VOC had mutations which confer reduced susceptibility to current vaccines and potential to evade detection by diagnostic tests such as RT-PCR. This work provides a model for WGS surveillance-based approaches to inform emerging and circulating SARS-CoV-2 diversity in Tanzania and elsewhere, and we advocate for periodic evaluation of performance characteristics of the RT-PCR tests in diagnosing rapidly mutating SARS-CoV-2 strains.

## 6. Study strengths and limitations

In addition to the ARTIC nCoV-2019. sequencing protocol v3 (LoCost) (12), we added a step of performing gel electrophoresis immediately after multiplex PCR to visualize presence of amplicons. In turn, more than a half of the individual samples had faint or no bands suggesting RNA degradation after collection or transportation. The limited sample size and absence of prior SARS-CoV-2 sequences from Tanzania precluded detailed phylodynamic analysis. Without a larger spatio-temporally distributed dataset, it was difficult to make inferences on the provenance, transmission dynamics, and in particular distribution of variants–whether novel, concerning, or otherwise–in the genome population. Another limitation was the moderate to poor genome coverage attained for a number of samples. Twelve sequences had coverage in the range 80–90% (24,000–26,999 non-N bases), and two were just below 80%. The major cause for this was amplicon drop out, which was partly remedied by adding primer pair 74_alt0 to the ARTIC V3 primer pool. Though most of the significant regions of the genomes were covered, such as the S gene and the sites that define the VOC assignments, SNPs located on the unknown parts of genomes cannot contribute information to phylogenetic analysis, hence lead to a less precise placement on the global phylogenetic tree. Finally, the study lacked complete demographic and clinical data that would have strengthened the interpretation of genomic data for SARS-CoV-2 isolates which was a function of using archived samples. Despite these limitations, the processes developed identified steps for improving data completeness and linkage to conventional epidemiological resources that will position WGS as a service to the Tanzanian scientific community.

## Data availability statement

The datasets presented in this study can be found in online repositories. The names of the repository and accession numbers can be found below: GenBank, OP236812–OP236838. The sequence files can also be found in GISAID with accession numbers EPI_ISL_16131912–EPI_ISL_16131937.

## Ethics statement

The studies involving human participants were reviewed and approved by College Research Ethics Review Committee, Kilimanjaro Christian Medical University College. Written informed consent for participation was not required for this study in accordance with the national legislation and the institutional requirements.

## Author contributions

SRM: conceptualization, data curation, formal analysis, funding acquisition, investigation, methodology, resources, validation, visualization, writing – original draft, and writing – review and editing. MvZ: data curation, formal analysis, investigation, methodology, resources, validation, visualization, and writing – review and editing. CCK: investigation, methodology, resources, validation, visualization, and writing – review and editing. PMM: validation, visualization, and writing – review and editing. ANM, ASM, and RMK: resources, validation and writing – review and editing. TBS: data curation, validation, visualization, and writing – review and editing. GSK: conceptualization, validation, and writing – review and editing. GG: methodology, resources, and writing – review and editing. SKH: validation and writing – review and editing. JOC: conceptualization, supervision, methodology, validation, and writing – review and editing. SGM: conceptualization, funding acquisition, investigation, methodology, resources, validation, visualization, supervision, and writing – review and editing. All authors contributed to the article and approved the submitted version.

## Supplementary material

Supplementary File 1 Excel spread sheet containing NextClade output and Pangolin output.

Supplementary File 2 A zipped folder containing json files for the time-resolved maximum likelihood phylogenies of Delta, Beta, and Eta variants that can be visualized using auspice web-based tool available at *https://auspice.us/?d=tree&p=full.* Sub-sampling of global reference genomes for all the variants was done from December 2019 to November 2021. The Delta variant file shows a phylogenetic tree of Delta variant combining 4 genomes from Tanzania (blue tips) and 2,591 global reference genomes (yellow). The Beta variant file shows a phylogeny of Beta variant combining 22 genomes from Tanzania (blue tips) and 1,845 global reference genomes (yellow). The Eta variant file shows a phylogeny of Eta variant combining 2 genomes from Tanzania (blue tips) and 998 global reference genomes (yellow).
